# Prediction of high-flow nasal cannula failure in patients with sepsis by a preliminary risk stratification model based on the ROX index

**DOI:** 10.1186/s12879-026-14135-3

**Published:** 2026-08-03

**Authors:** Yuanwen Ye, Feifei Li, Liangen Lin, Linglong Chen, Baohua Yang, Xiaowu Wei

**Affiliations:** 1 Department of Emergency, The Wenzhou Third Clinical Institute Affiliated To Wenzhou Medical University, Wenzhou People’s Hospital, Wenzhou, 325000 China; 2 Department of Anesthesia and Surgery, The Wenzhou Third Clinical Institute Affiliated To Wenzhou Medical University, Wenzhou People’s Hospital, Wenzhou, 325000 China

**Keywords:** HFNC, Sepsis, ROX index, Preliminary risk stratification model

## Abstract

**Background:**

High-Flow Nasal Cannula (HFNC) carries the risk of delayed intubation and invasive mechanical ventilation (IMV), often associated with adverse outcomes. Therefore, it is crucial to predict the risk of HFNC failure as early as possible.

**Objective:**

To explore the value of the Respiratory Rate-Oxygenation (ROX) index and a Preliminary Risk Stratification Model based on the ROX Index for prediction of HFNC failure in patients with sepsis.

**Methods:**

This retrospective study was based on the Medical Information Mart for Intensive Care (MIMIC) database. Independent risk factors for HFNC failure were explored using Least Absolute Shrinkage and Selection Operator (LASSO) and multivariable logistic regression analyses, with discrimination shown using Receiver Operating Characteristic Curves (ROC). To evaluate the discriminatory ability of the ROX index at different time points, we performed Landmark Analysis. The relationship between the ROX index and the risk of HFNC failure was confirmed through logistic proportional hazards model. Kaplan-Meier (K-M) curves were employed to illustrate the association between the ROX index and the likelihood of HFNC failure. Additional subgroup analyses were conducted to further substantiate the robustness of the findings. Finally, a Preliminary risk stratification model for predicting HFNC failure was established based on the ROX index, with ROC curves used to demonstrate its discriminatory power.

**Results:**

A total of 316 sepsis patients who met the criteria were included, of whom 116 patients failed HFNC and required intubation and IMV. LASSO and multivariable logistic analyses demonstrated the ROX index as an independent risk factor for HFNC failure, with an area under the ROC curve of 0.721 (0.658–0.784) after 12 h of HFNC use, indicating good predictive value. Logistic proportional hazards model and K-M analysis demonstrated that patients in the low ROX group (based on the established cutoff value) exhibited a significantly elevated risk of failure of HFNC therapy. This finding was consistent with the results of subgroup analyses. Based on the ROX index, a Preliminary risk stratification model with a total score of approximately 16 points was established by combining Glasgow Coma Scale (GCS), Body Mass Index (BMI), and flow rate, and the area under the ROC curve was 0.801 (0.7516–0.8509), indicating better predictive value.

**Conclusion:**

The ROX index has good predictive value for HFNC failure in sepsis patients, and the Preliminary risk stratification model established based on the ROX index shows better predictive potential, but requires prospective external validation.

**Supplementary Information:**

The online version contains supplementary material available at https://doi.org/10.1186/s12879-026-14135-3.

## Introduction

Sepsis poses a significant threat to patients’ lives [1]. In addition to hemodynamic resuscitation and early antibiotic administration, the use of oxygen therapy is crucial for sepsis patients experiencing hypoxemia in the initial stages of treatment [2]. High-Flow Nasal Cannula (HFNC), a significant noninvasive technique for delivering high concentrations of oxygen, has gained widespread popularity, particularly as a preferred method of noninvasive oxygen therapy for hypoxemia in critically ill patients [3].

HFNC provides stable oxygen levels at a flow rate of 50–60 L/min while applying minimal positive end-expiratory pressure in the upper airway [4]. Electrical impedance tomography studies have further demonstrated that HFNC can effectively improve local ventilation distribution, increase end-expiratory lung volume (EELV), and enhance lung compliance [5], thereby significantly reducing patients’ respiratory effort and the need for endotracheal intubation and IMV [6–7].

However, in the management of HFNC, deciding when to switch from HFNC to IMV remains a challenging decision. It is noted that although HFNC may prevent the need for IMV in some patients, it may adversely impact the outcomes of others by unduly delaying IMV initiation [8]. As IMV initiation is delayed, the associated risk further increases [9]. Therefore, identifying and describing accurate early predictors of HFNC success and failure are of particular importance.

The Respiratory Rate-Oxygenation (ROX) index is the ratio of pulse oxygen saturation (SpO_2_)/fraction of inspired oxygen (FiO_2_) to the respiratory rate (RR) [10]. Because it requires very few parameters and can be obtained at the bedside, the ROX index has great application potential. ROX has been shown to be valuable in predicting the success and failure of HFNC in patients with acute hypoxemic respiratory failure [11, 12], pneumonia patients with hypoxemic respiratory failure [9, 10], and other conditions. However, its value in sepsis, a condition characterized by tissue hypoxia, remains unclear. Therefore, it is of particular interest to explore the value of ROX and a Preliminary Risk Stratification Model in predicting the risk of HFNC failure in patients with sepsis.

## Methods

### Data source

This retrospective analytic study was based on data obtained exclusively from the MIMIC-IV database. MIMIC-IV is an updated version of MIMIC-III, derived from the critical care database at Beth Israel Deaconess Medical Center (BIDMC) in the United States. This database includes data from 2008 to 2019, encompassing over 200,000 emergency admissions and more than 60,000 intensive care unit (ICU) admissions. The data include demographics, laboratory tests, imaging, vital signs, diagnostic and medical intervention records, and more. Researchers completed the “Protecting Human Research Participants” course and obtained ethical approval from BIDMC and the Massachusetts Institute of Technology (MIT) (Record ID: 43546933).

### Inclusion criteria

1. Sepsis 3.0 [2]. Sepsis is characterized as a life-threatening condition resulting from organ dysfunction due to a dysregulated host response to infection. For clinical application, organ dysfunction can be operationalized by an increase of 2 points or more in the Sequential [Sepsis-related] Organ Failure Assessment (SOFA) score.
2. Records of HFNC usage.
3. Use of HFNC as ventilatory support before IMV.

### Exclusion criteria

1. HFNC for less than 2 h.
2. Records of NIV use before intubation and IMV, i.e., HFNC-NIV-IMV (NIV use affects the ROX index).
3. Tracheostomy performed before admission.
4. Minors (age < 18).
5. Multiple hospitalizations, excluding the first hospitalization.
6. Hospital stay less than 48 h.
7. Conditions with high risk of hypercapnia (Chronic Obstructive Pulmonary Disease).
8. Lack of ROX-related data.

Rationale for excluding patients with HFNC duration < 2 h: Patients who received HFNC for less than 2 h were excluded because they typically experienced rapid clinical deterioration that rendered HFNC unsuitable from the outset, necessitating immediate intubation and IMV. In such scenarios, no meaningful “prediction” window exists. Therefore, excluding these patients helps focus the analysis on the target population that can potentially benefit from HFNC and requires dynamic risk assessment.

The sample size was determined by the availability of eligible cases in the MIMIC-IV database meeting the prespecified inclusion and exclusion criteria during the study period. No formal sample size calculation was performed prior to data extraction, as this was a retrospective exploratory prognostic study. The final sample of 316 patients (116 events) yielded an events-per-variable (EPV) ratio of approximately 29:1 for the 4 predictors included in the final model, exceeding the recommended minimum of 10 events per candidate predictor.

#### Research methods

Data extraction was conducted using PostgreSQL. The variables included demographic information, vital signs, critical illness scoring systems, clinical treatment information, and clinical outcomes. Demographic information included age, gender, ethnicity, and BMI, calculated as weight (kg) divided by height squared (m²). Vital signs included systolic blood pressure (SBP), diastolic blood pressure (DBP), mean arterial pressure (MAP), heart rate (HR), respiratory rate (RR), and pulse oxygen saturation (SpO_2_). In the analysis, the first time vital sign values measured after the use of HFNC were utilized as indicators of disease progression. Critical illness scoring systems included the Charlson Comorbidity Index (CCI), GCS, Sequential Organ Failure Assessment (SOFA), Simplified Acute Physiology Score II (SAPS II), and Model for End-Stage Liver Disease (MELD) score, utilized only the most severe values within the initial 24 h of HFNC use for analysis. Clinical treatment information included continuous renal replacement therapy (CRRT), vasopressor use, length of hospital stay, length of ICU stay, and duration and parameters (flow rate) of HFNC.

The primary research variable was the ROX index, defined as the ratio of pulse oxygen saturation (SpO_2_) to the fraction of inspired oxygen (FiO_2_) and respiratory rate (RR). The ROX index was recorded at the initiation of HFNC (ROX-initial) and at 2 h (ROX-2 h), 6 h (ROX-6 h), and 12 h (ROX-12 h) after initiation, using SpO_2_, FiO_2_, and RR at the same time point for each measurement, with a permissible time difference of 30 min.

  

$$\:ROX\:index\hspace{0.17em}=\hspace{0.17em}Sp{O}_{2}\:/\:(FiO2\:\times\:\:RR)$$

The primary outcome was HFNC success and failure, defined as success if HFNC was continued, downgrading to a lower level of oxygen support, or complete weaning from oxygen support, and as failure if intubation and IMV was required. (During the exclusion process, only a small number of patients received NIV after HFNC failure (*n* = 43), and most of them eventually required intubation and IMV. Therefore, we defined HFNC failure as the need for intubation and IMV, which is a more clinically relevant and objective endpoint).

To mitigate potential bias stemming from a substantial amount of missing data, variables with more than 30% missing values were excluded from subsequent analyses. Correspondingly, for variables with less than 30% missing values, missing data were handled using multiple imputation (MI) with multivariate normal regression (MVN), generating 20 imputed datasets. The proportion of missingness for each variable is reported in Table S7.

#### Statistical methods

The normality of the variables was assessed using Kolmogorov-Smirnov test. Continuous parametric variables were reported as mean (standard deviation), whereas non-parametric variables were expressed as median (interquartile range). For comparisons, the Unpaired Student’s t-test was employed for parametric variables, while the Mann–Whitney U-test was used for non-parametric variables. Categorical variables were analyzed using the χ2 test or Fisher’s exact test. Significant variables from LASSO regression analysis were included in a multivariate logistic regression to identify the independent risk factors associated with HFNC failure. The predictive value of the ROX index for HFNC failure was assessed by plotting ROC curves.

To evaluate the dynamic predictive performance of the ROX index at different time points during the course of HFNC therapy, this study conducted two Landmark analyses at 6 h and 12 h after HFNC initiation, respectively. At each Landmark time point, the analysis included only patients who were still receiving HFNC and had not undergone endotracheal intubation and IMV at that time, thereby excluding patients who had already experienced HFNC failure before the Landmark time point. This design was intended to simulate real-world clinical practice, where risk assessment can only be performed while the patient remains on HFNC, and to evaluate whether the predictive performance of the ROX index improves with longer duration of HFNC use. The optimal cutoff values for ROX-6 h and ROX-12 h in the Landmark analyses were determined using Youden’s index from the ROC curves.

The association between ROX index and HFNC failure risk was verified using a logistic proportional hazards model, presenting results as odds ratios (OR) and 95% confidence intervals (CI). Survival curves were plotted using the K-M method, and log-rank tests were used to compare differences between groups, with a series of subgroup analyses performed to validate the robustness of the results.

#### Model development and internal validation procedures

The Preliminary Risk Stratification Model was developed following a five-step approach.

*Step 1 – Candidate predictor selection.* Based on clinical relevance and previous literature, 17 candidate variables were prespecified, including demographic characteristics (age, gender, BMI, ethnicity), vital signs (HR, SBP, DBP, MAP, RR, SpO₂, Flow rate-initial), illness severity scores (SOFA, GCS, SAPS II, MELD, CCI), and the ROX index.

*Step 2 – Variable shrinkage and selection.* LASSO regression with 10-fold cross-validation was applied to the 17 candidate variables to select predictors with non-zero coefficients. The optimal penalty parameter λ was selected using the minimum partial likelihood deviance criterion (1-standard-error rule). This step identified 8 predictors with non-zero coefficients.

*Step 3 – Multivariable logistic regression.* The 8 LASSO-selected variables were entered into a multivariable logistic regression model with backward stepwise elimination (retention criterion *P* < 0.05). This yielded 4 independent predictors: ROX-initial, GCS, BMI, and Flow rate.

*Step 4 – Score derivation.* Points for each predictor were assigned proportionally to the absolute values of the regression coefficients, rounded to the nearest integer, following the method of Sullivan et al. [13].

*Step 5 – Internal validation.* The performance of the final model was internally validated using bootstrap resampling with 1,000 iterations. Model discrimination was evaluated using the AUC, with optimism-corrected AUC reported. Model calibration was assessed using calibration plots with smoothed loess curves and the Hosmer-Lemeshow goodness-of-fit test, where *P* > 0.05 indicates acceptable calibration.

This prognostic study was reported in accordance with the Transparent Reporting of a multivariable prediction model for Individual Prognosis Or Diagnosis (TRIPOD) statement. A completed TRIPOD checklist is provided in the supplementary materials.

#### Sensitivity analysis

To assess the robustness of our findings regarding NIV exclusion, we performed a sensitivity analysis by reincluding the 43 patients who received NIV before intubation and IMV (expanded cohort, *n* = 359). HFNC failure was redefined as escalation to either NIV or IMV. The discriminative ability of the ROX index was re-evaluated using the AUC in this expanded cohort.

To assess whether imbalances in baseline characteristics affected the robustness of our results, we performed propensity score matching (PSM) as a sensitivity analysis. We used 1:1 PSM to adjust for baseline characteristics, including demographic information (age, gender, ethnicity, BMI), vital signs (SBP, DBP, MAP, HR, RR, SpO₂, flow rate), and Critical illness scoring systems (CCI, GCS, SOFA, SAPS II, MELD). In the PSM cohort, the discriminatory ability of the ROX index was re-evaluated using AUC.

All statistical analyses were conducted using SPSS 26.0 and R4.4.1 software, with a significance level of *P* < 0.05.

## Results

### General characteristics of the population included

The MIMIC-IV database contains 35,010 patients with sepsis. After rigorous screening based on inclusion and exclusion criteria, 316 patients were included in this study. For details of the inclusion and exclusion criteria, refer to Figure S1. Patients were categorized into two groups based on HFNC outcomes: the success group (200 patients, 63.3%) and the failure group (116 patients, 36.7%).

**Fig. 1:**
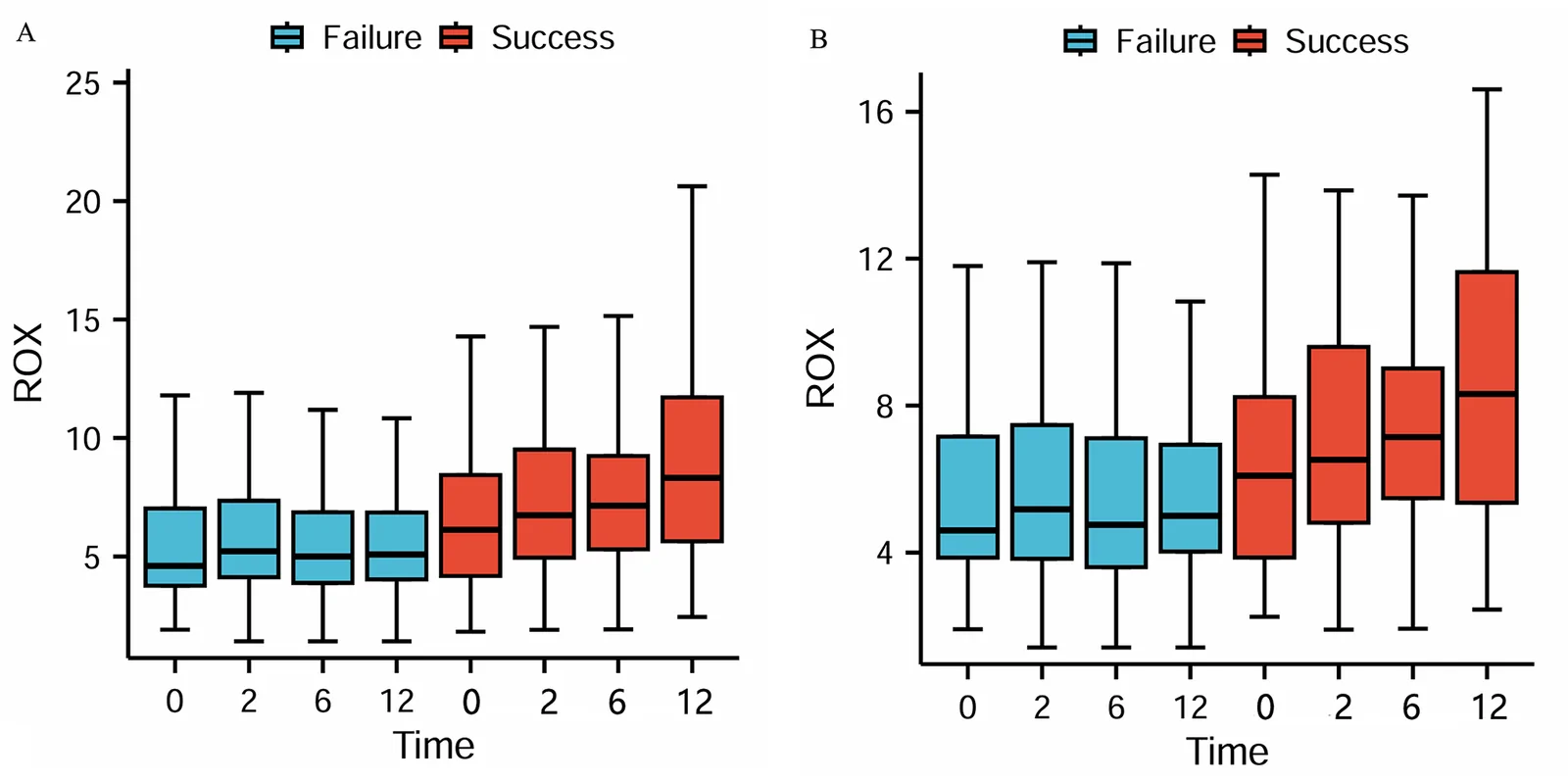
ROX trend analysis chart (**A**: After PSM; **B**: Before PSM)

In the original cohort, the ROX index was significantly higher in the HFNC success group compared with the failure group (6.13 [4.15, 8.43] vs. 4.60 [3.74, 7.08], *p* = 0.001). In the PSM cohort, a higher ROX index was observed in the HFNC success group, but the difference was not statistically significant (6.09 [3.87, 8.23] vs. 4.60 [3.86, 7.16], *p* = 0.088), see Table 1 for details. Notably, in the successful group, the ROX index gradually increased from a baseline value of 6.13 (interquartile range 4.15–8.43) to 8.32 (interquartile range 5.61–11.82) at 12 h, with statistically significant differences between time points (trend P-value < 0.001); In contrast, in the failure group, the ROX index rose only slightly from a baseline value of 4.60 (interquartile range 3.74–7.08) to 5.08 at 12 h (interquartile range 4.01–6.90), with no statistically significant change (trend P-value = 0.337). The same trend was observed in the PSM cohort; see Fig. 1.

**Table 1:** Baseline characteristics of the patients with sepsis comparing success and failure group

Characteristics	Before PSM					After PSM				
	Total (*n* = 316)	Success Group (*n* = 200)	Failure Group (*n* = 116)	Statistic	*P*	Total (*n* = 160)	Success Group (*n* = 80)	Failure Group (*n* = 80)	Statistic	*P*
**Demographic**										
Age, year (median [IQR])	67.2(57.2, 79.7)	71 (59, 82)	63 (55, 77)	-2.638	0.008	64.7 (55.2, 80.1)	65.7 (54. 5, 82.3)	63.6 (57.0, 79.1)	0.36	0.719
Male, gender (n(%))	188(59.5)	109 (54.5)	79 (68.1)	5.638	0.018	102 (63.8)	52 (65.0)	50 (62.5)	0.11	0.742
Ethnicity, White (n(%))	232(73.4)	150 (75.0)	82 (70.7)	0.699	0.403	110 (68.8)	54 (67.5)	56 (70.0)	0.12	0.733
BMI, kg/m^2^ (median [IQR])	26.3(23.5, 30.3)	25.9 (23.5, 29.5)	27.3 (23.4, 31.8)	-1.912	0.056	26.8 (24.0, 31.5)	26.7 (24.3, 31.2)	26.9 (23.3, 31.6)	-0.36	0.721
**Vital sign**										
HR, bpm (median [IQR])	95.0(82.0, 109.0)	94 (82, 108)	97 (82, 115)	-1.092	0.275	97.5 (82.0, 108.0)	97.5 (82.0, 108.0)	97.0 (79.3, 111.5)	-0.11	0.916
SBP, mmHg (median [IQR])	119.0(106.0, 134.0)	118 (106, 131)	121 (106, 143)	-0.960	0.337	119.0 (108.0, 136.3)	119.0 (110.8, 133.3)	121.0 (104.8, 143.3)	-0.06	0.955
DBP, mmHg (median [IQR])	66.0(54.0, 77.0)	66 (54, 77)	67 (56, 78)	-0.429	0.668	65.5 (54.8, 78.3)	63.5 (54.0, 77.9)	67.0 (56.8, 78.3)	-0.61	0.545
MBP, mmHg (median [IQR])	80.0(70.0, 92.2)	79 (69, 92)	83 (71, 94)	-1.347	0.178	80.5 (70.0, 94.0)	78.0 (68.8, 94.0)	83.0 (72.0, 94.0)	0.77	0.443
RR, bpm (median [IQR])	24.0(20.0, 29.0)	23 (20, 28)	25 (21, 29)	-1.654	0.098	25.0 (20.0, 29.0)	24.0 (20.0, 29.3)	25.5 (21.0, 29.0)	-0.76	0.448
SpO_2_ (median [IQR])	95.0(92.0, 97.0)	95 (92, 97)	94 (91, 97)	-1.115	0.265	94.0 (92.0, 97.0)	94.0 (92.0, 96.0)	94.0 (91.0, 97.0)	-0.17	0.868
Flow rate-initial, L/min (median [IQR])	30.0(15.0, 40.0)	30 (15, 40)	40 (25, 40)	-4.115	< 0.001	35.0 (25.0, 40.0)	35.0 (18.8, 40.0)	40.0 (25.0, 40.0)	-1.07	0.287
**Critical illness scoring systems**										
SOFA-first-day (median [IQR])	6.0(4.0, 8.0)	5.0 (3.0, 7.0)	7.0 (5.0, 11.0)	-5.806	< 0.001	6.5 (4.0, 9.0)	7.0 (4.0, 8.0)	6.0 (4.0, 9.3)	-0.03	0.974
GCS-first-day (median [IQR])	13.0(9.0, 14.0)	14.0 (12.0, 15.0)	10.0 (6.0, 14.0)	-6.827	< 0.001	13.0 (9.0, 14.0)	13.0 (9.0, 14.0)	12.50 (9.0, 14.0)	-0.73	0.464
SAPS-II-first-day (median [IQR])	36.5(29.0, 45.2)	36.0 (27.0, 43.0)	39.0 (30.0, 51.0)	-2.484	0.013	37.0 (29.0, 46.0)	37.5 (26.5, 46.0)	36.0 (29.0, 46.0)	0.18	0.860
Meld-first-day (median [IQR])	13.0(9.0, 20.0)	12.5 (9.0, 18.0)	15.0 (10.0, 23.8)	-2.743	0.006	14.0 (10.0, 21.0)	13.5 (10.0, 21.0)	14.0 (9.8, 20.3)	-0.34	0.733
Charlson Comorbidity Index (median [IQR])	6.0(4.0, 8.0)	6.0 (4.0, 9.0)	5.0 (4.0, 7.0)	-2.481	0.013	6.0 (4.0, 8.0)	5.5 (3.8, 8.0)	6.0 (4.0, 8.0)	-0.24	0.812
**ROX**										
ROX-initial (median [IQR])	5.70(3.92, 8.08)	6.13 (4.15, 8.43)	4.60 (3.74, 7.08)	− 0.3.351	0.001	5.13 (3.86, 7.71)	6.09 (3.87, 8.23)	4.60 (3.86, 7.16)	-1.70	0.088
ROX-2 h (median [IQR])	6.14(4.62, 8.61)	6.74 (4.95, 9.55)	5.22 (4.13, 7.36)	-3.752	< 0.001	5.74 (4.45, 8.68)	6.52 (4.81, 9.60)	5.18 (3.83, 7.47)	-2.60	0.009
ROX-6 h (median [IQR])	6.39(4.60, 8.69)	7.14 (5.25, 9.25)	5.00 (3.83, 6.92)	-5.728	< 0.001	6.10 (4.11, 8.70)	7.14 (5.48, 9.01)	4.75 (3.60, 7.11)	-3.96	< 0.001
ROX-12 h (median [IQR])	6.96(4.79, 10.3)	8.32 (5.61, 11.82)	5.08 (4.01, 6.90)	-7.720	< 0.001	6.47 (4.49, 9.51)	8.32 (5.36, 11.64)	5.00 (4.03, 6.93)	5.20	< 0.001
**Clinical treatment information**										
CRRT (n(%))	40(12.7)	12 (6.0)	28 (24.1)	21.846	< 0.001	23 (14.38)	8 (10.00)	15 (18.75)	2.49	0.115
Vasopressor use (n(%))	135(42.7)	45 (22.5)	90 (77.6)	91.043	< 0.001	85 (53.12)	26 (32.50)	59 (73.75)	27.33	< 0.001
Length of hospital stay, day (median [IQR])	13.7(8.08, 22.0)	11.0 (7.0, 18.0)	20.0 (13.0, 33.0)	-7.026	< 0.001	15.7 (8.8, 23.9)	11.5 (6.9, 19.8)	19.1 (12.2, 30.4)	-4.40	< 0.001
Length of ICU stay, day (median [IQR])	5.8(3.4, 11.6)	4.0 (3.0, 6.0)	13.0 (9.0, 19.0)	-10.729	< 0.001	7.7 (3.9, 12.6)	4.2 (2.9, 6.3)	12. (8.3, 16.6)	-7.43	< 0.001
Duration of HFNC, hour (median [IQR])	25.6(10.8, 52.1)	31.0 (14.5, 66.3)	14.8 (7.0, 35.2)	-4.768	< 0.001	22.4 (9.0, 47.6)	31.5 (14.8, 64.4)	16.0 (7.0, 36.0)	-3.26	0.001

### Association between delayed intubation and hospital mortality

**Fig. 2:**
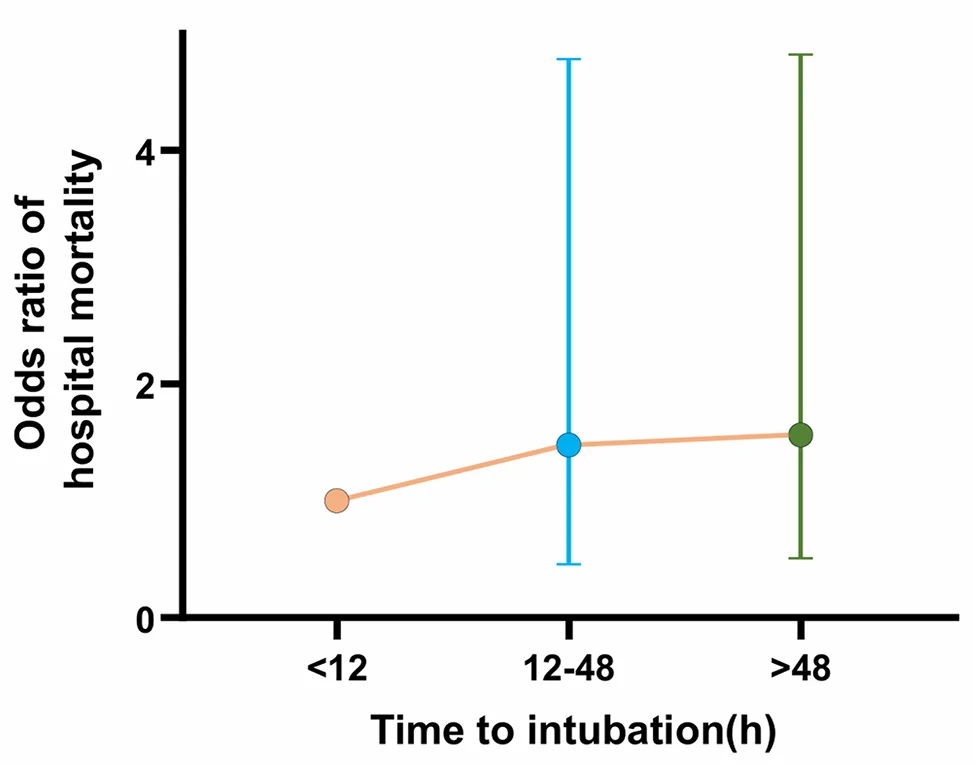
Odds ratio of hospital mortality according to the time to intubation in patients who failed on HFNC

To clarify when it is most appropriate to assess the risk of HFNC failure, we used the first 12 h of HFNC treatment as a reference to determine mortality rates within different time phases. Among patients who failed HFNC, compared to those intubated within ≤ 12 h, patients intubated between 12 and 48 h and > 48 h had numerically higher in-hospital mortality (1.476-fold and 1.566-fold, respectively), but these differences did not reach statistical significance (*P* > 0.05). As illustrated in Fig. 2.

### Explore risk factors for HFNC failure

**Fig. 3:**
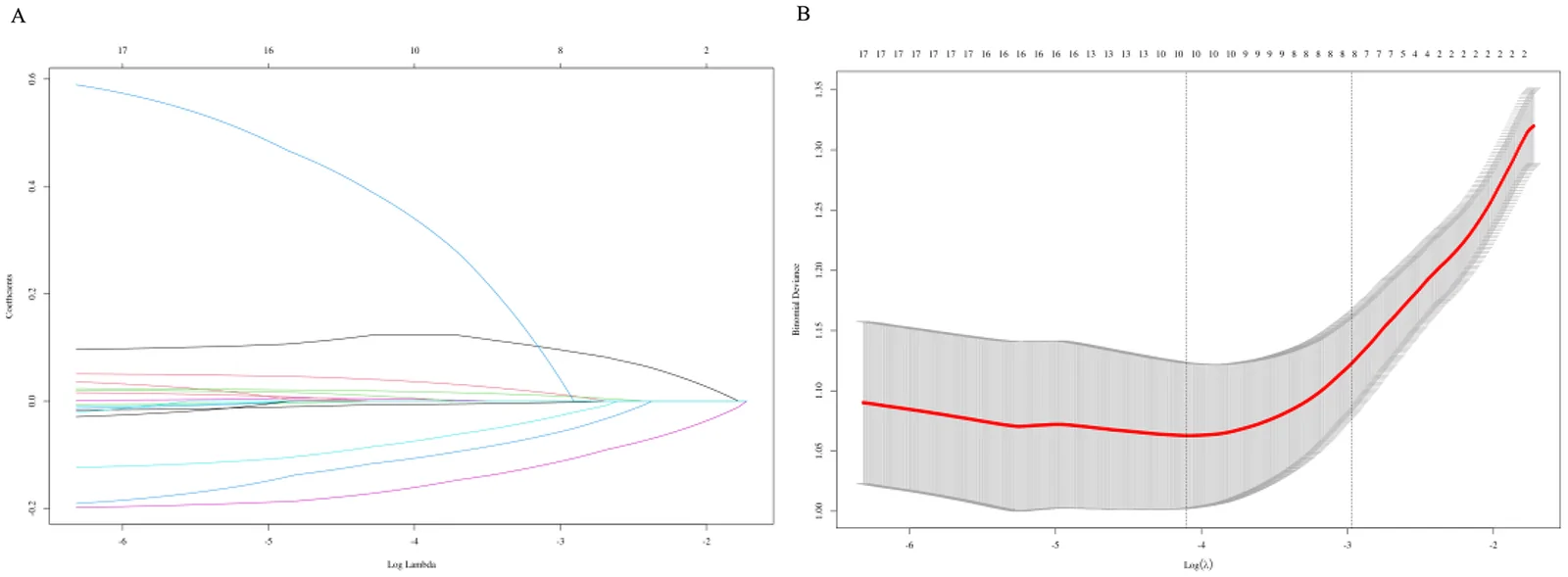
Variable selection for the regression model (**A**: 10-fold cross-validation to select the optimal λ; **B**: Derivation of the optimal λ to select 8 variables with non-zero coefficients)

LASSO regression identified 8 predictors with non-zero coefficients from the initial 17 variables: Age, BMI, Flow rate-initial, ROX, CCI, GCS, SOFA, and Gender (see Fig. 3). Multivariate logistic regression analysis revealed that Flow rate-initial, ROX, GCS, and BMI were independent predictors of HFNC failure in patients with sepsis (all *P* < 0.05), as shown in Table S1.

### ROX index predicts value of HFNC failures

**Fig. 4:**
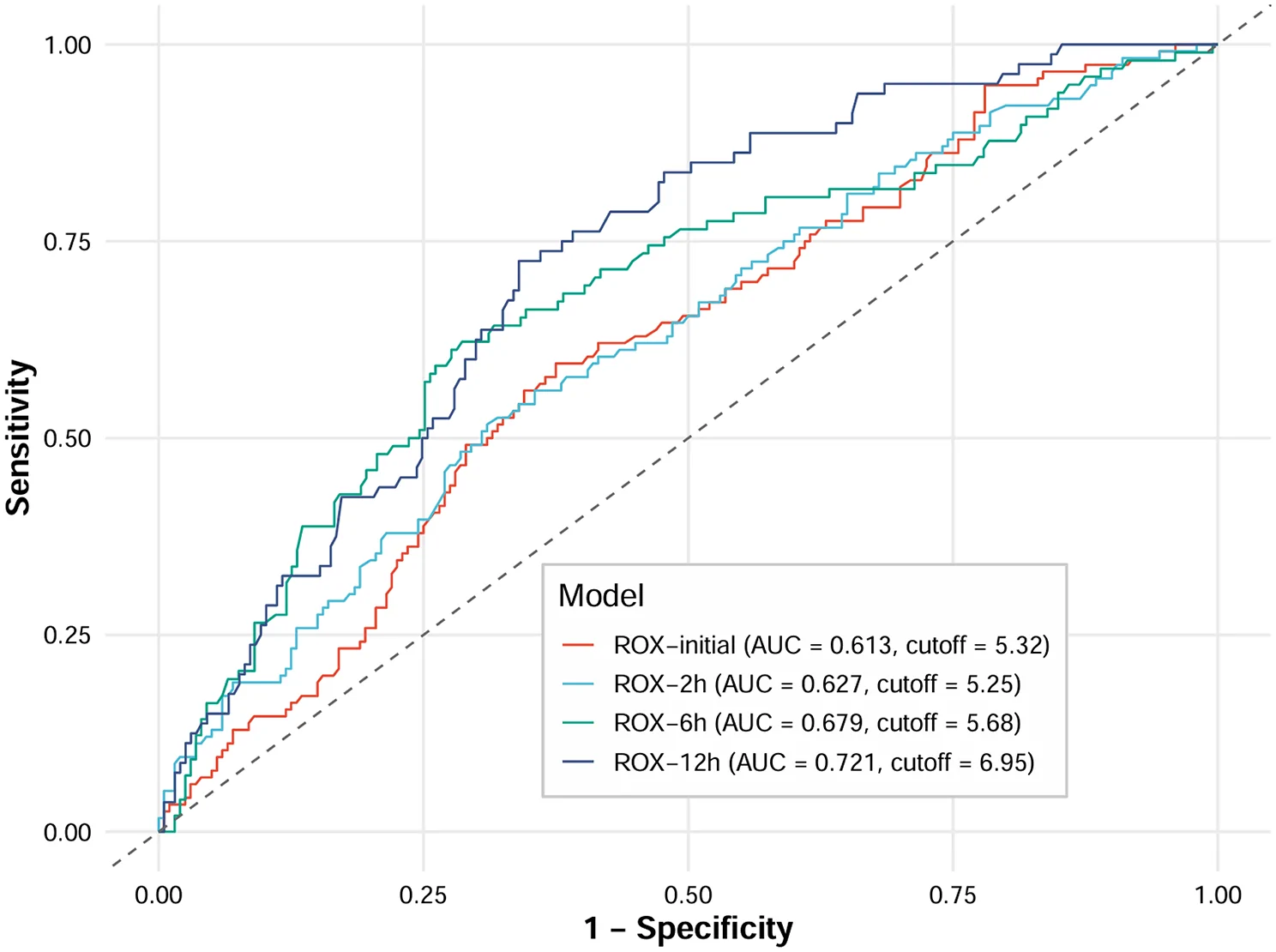
ROC curve of ROX

The ROX index demonstrated an AUC of 0.613 for the initial identification of HFNC failure. As the duration of HFNC use extended, the index’s ability to discern subsequent treatment failures became increasingly apparent among patients who continued to tolerate HFNC. Specifically, the AUC values were 0.627 at 2 h, 0.679 at 6 h, and 0.721 at 12 h, as shown in Fig. 4. At the optimal cutoff value of 6.95 (ROX-12 h cutoff of 6.95), the sensitivity for identifying HFNC failure was 72.5% and the specificity was 66.0%, as shown in Table S2. In the low-risk zone (ROX-12 h > 8.05), sensitivity exceeds 80%, suggesting that continued HFNC therapy may be considered; in the high-risk zone (ROX-12 h < 5.22), specificity exceeds 80%, requiring high vigilance and readiness for intubation.

To further validate the predictive value of the ROX index, we divided the patients into low ROX (< 6.95) and high ROX (≥ 6.95) groups using the optimal cutoff value of 6.95 based on ROX-12 h (ROX-12 h cutoff of 6.95), and applied logistic regression modeling to explore the relationship between ROX and HFNC failure. Using the high ROX group as the reference, Model I showed that patients in the low ROX group had a significantly increased risk of HFNC failure (low ROX group: OR = 5.115; 95% CI: 2.886 ~ 9.068; *P* < 0.05). After adjusting for demographic data such as age and gender, Model II showed similar results (low ROX group: OR = 4.968; 95% CI: 2.780 ~ 8.876; *P* < 0.05) (see Table S3). After further adjusting for confounders such as age, gender, and vital signs, Model III showed similar results (low ROX group: OR = 5.729; 95% CI: 3.079 ~ 10.661; *P* < 0.05) (see Table S3). The results of Model IV showed that ROX was an independent risk factor for HFNC failure, with the low ROX group having an increased risk compared to the high ROX group (OR = 5.780; 95% CI: 2.839 ~ 11.771; *P* < 0.05) (see Table S3).

K-M analysis similarly showed that patients in the low ROX group had a significantly increased risk of HFNC failure. The cumulative probability of HFNC success for different ROX groups are displayed in the K-M plot in Figure S2 (Log-rank χ² = 18.54, P = < 0.001). Furthermore, as shown in Table S4, the association between ROX and HFNC failure was consistent across most strata, except for some subgroups with smaller sample sizes.

### Preliminary risk stratification model predicts value of HFNC failures

We established a Preliminary risk stratification model by combining the four independent risk factors of BMI, flow rate, GCS, and ROX. The detailed development process is shown in Table S5. This Preliminary risk stratification model has a total of 16 points, with maximum scores of 3 for BMI, 6 for flow rate, 5 for GCS, and 2 for ROX, as shown in Table 2. Of note, the ROX index used in this model refers to the initial value at HFNC initiation, with an optimal cutoff of 5.32 (ROX-initial cutoff of 5.32). Individual scores for BMI, flow rate, GCS, and ROX were calculated separately, and a total score was obtained to assess the specific risk of HFNC failure, as shown in Table S6.

**Fig. 5:**
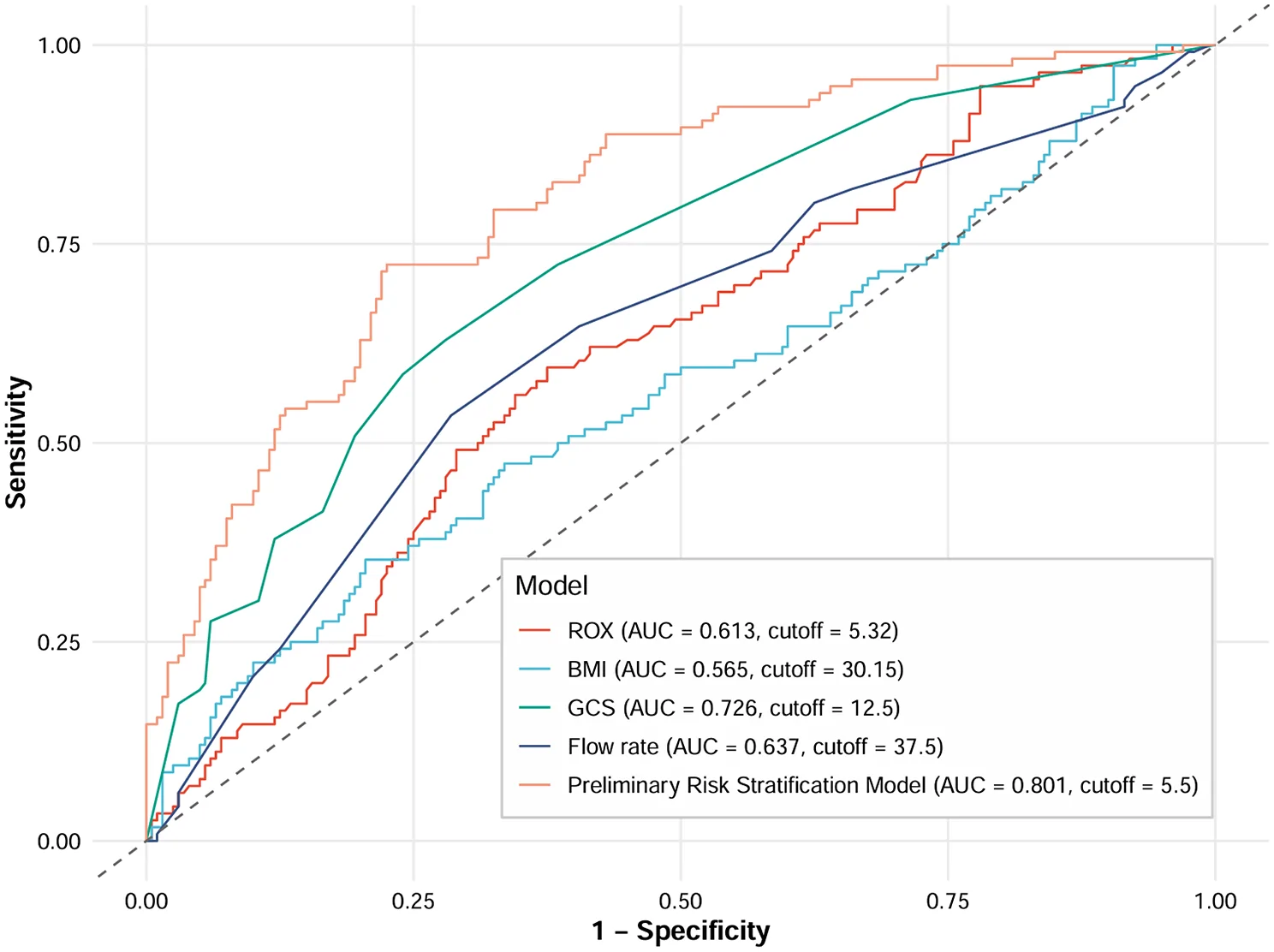
ROC curve of the preliminary risk stratification model

This Preliminary risk stratification model demonstrated good discriminatory performance, with an AUC of 0.801 (95% CI: 0.7516–0.8509) for the original model, as shown in Fig. 5. After 1,000 bootstrap repetitions, the C-statistic (adjusted AUC) was 0.7824 (95% CI: 0.7341–0.83), and the calibration plot showed good agreement between predicted and observed probabilities (Hosmer-Lemeshow test, *P* = 0.409), indicating good stability, as shown in Figure S3. At the optimal cutoff value of 5.5, the sensitivity for identifying HFNC failure was 77.5%, and the specificity was 72.4%, as shown in Table S2.

**Table 2:** Preliminary risk stratification model based on the ROX Index

Factor	Categories	Score
GCS		
	13–15	0
	9–12	2
	6–8	4
	3–5	6
BMI		
	< 18.5	-1
	18.5–24.9	0
	25–30	1
	> 30	3
Flow rate		
	1–25	0
	26–50	2
	51–75	3
	76–100	5
ROX-initial		
	≥ 5.32	0
	< 5.32	2

Note: The ROX index in this table is the initial value at HFNC initiation (ROX-initial cutoff of 5.32). This cutoff differs from the 12-hour ROX cutoff (ROX-12 h cutoff of 6.95)

### Sensitivity analysis

To assess the robustness of our findings regarding NIV exclusion, we reincluded the 43 patients (expanded cohort, *n* = 359) and redefined the endpoint as “escalation to either NIV or IMV.” In the expanded cohort (*n* = 359), the HFNC failure rate increased from 36.7% (116/316) in the original cohort to 44.3% (159/359). Under this definition, the ROX index demonstrated an AUC of 0.627 for the initial identification of HFNC failure, which improved to 0.629 at 2 h and 0.690 at 6 h. The AUC of ROX-12 h for predicting HFNC failure was 0.710 (95% CI: 0.652–0.768), which was close to that in the primary analysis (AUC = 0.721, 95% CI: 0.658–0.784), with a same optimal cutoff of approximately 6.95, as shown in Figure S4.

To assess whether imbalances in baseline characteristics affected the robustness of our results, we performed PSM as a sensitivity analysis to balance baseline characteristics between the success and failure groups. In the PSM cohort, all baseline characteristics were balanced (*P* > 0.05). The AUC of ROX-12 h for predicting HFNC failure was 0.707 (95% CI: 0.621–0.793), which was similar to the results of the primary analysis (AUC = 0.721, 95% CI: 0.658–0.784), with an optimal cutoff value of approximately 6.95, consistent with the value of 6.95 from the original cohort, as shown in Figure S5.

## Discussion

This study analyzed data from 316 patients with sepsis treated with HFNC to evaluate the predictive value of the ROX index for HFNC treatment failure. The results showed that the ROX index was higher in the successful treatment group, indicating that this index is an independent risk factor in addition to the GCS, airflow rate, and BMI. The ROX index effectively predicts HFNC treatment failure, and as the duration of HFNC use increases, its ability to identify subsequent treatment failure becomes increasingly evident among patients who continue to tolerate HFNC. A ROX index below 6.95 (ROX-12 h cutoff of 6.95) strongly suggests HFNC treatment failure.

The physiological rationale behind the ROX index is sound, as it integrates two key components of respiratory function: oxygenation efficiency (represented by the SpO₂/FiO₂ or S/F ratio) and the patient’s ventilatory demand (represented by respiratory rate) [9, 10]. Patients with treatment failure typically present with persistent worsening hypoxemia despite an increase in FiO₂, accompanied by a sustained or persistently elevated respiratory rate due to increased respiratory work; these two factors collectively drive a decrease in the ROX index. Our findings corroborate this mechanism, as the ROX index in the HFNC failure group was significantly lower than that in the successful treatment group. The predictive power of the ROX index is not static; as demonstrated by our data and the study by Ge et al., the index’s accuracy in identifying patients at risk of failure improves with prolonged HFNC use [14]. The initial ROX value provides only a snapshot, whereas the trajectory of the index—or values at later time points, such as 12 h—offers a more refined and reliable prognostic signal. This may be due to heterogeneity in patients’ early responses to HFNC; only when sustained physiological improvement fails to materialize after several hours can non-responders be reliably identified.

When comparing the specific thresholds of the ROX index and its predictive performance across different studies, significant heterogeneity emerged that warrants clinical interpretation. We derived a threshold of 6.95 (ROX-12 h cutoff of 6.95) for predicting HFNC failure; this value is closer to thresholds reported in broader acute hypoxic respiratory failure cohorts rather than the original threshold of 4.88 established by Roca et al. in a specific pneumonia cohort, which is widely cited [9, 10]. In our analysis, applying a cutoff value of 4.88 resulted in excessively low sensitivity; this finding is consistent with the results of Kansal et al., who observed that in their comprehensive ICU population, the optimal ROX cutoff values achieving 90% specificity at 2 h and 12 h were 5.92 and 7.40, respectively [15]. The study by Lun et al. further corroborates this variation; they reported an optimal 12-hour ROX cutoff of 5.626, but with a corresponding sensitivity of 88% and a specificity of only 41% [16, 17]. These differences in thresholds highlight that the ROX index is not a “one-size-fits-all” indicator. Its predictive performance is influenced by the patient population—including the etiology of respiratory failure (e.g., sepsis vs. uncomplicated pneumonia), the prevalence of immunocompromised states, and the severity of baseline disease. Sepsis, in particular, introduces confounding physiological variables such as hemodynamic changes and metabolic acidosis, which can drive tachypnea independently of pulmonary mechanisms, potentially requiring higher ROX cutoff values to maintain adequate predictive specificity, as observed in our study.

This study further suggests that, compared to relying solely on the ROX index, the use of a composite assessment strategy may improve predictive accuracy. We found that the GCS, initial HFNC flow rate, and BMI are also independent risk factors for HFNC failure. It has been reported that changes in flow rate can alter treatment outcomes by creating a sustained pressure effect in the airway, facilitating the clearance of dead space, increasing end-expiratory volume, and decreasing respiratory rate and work of breathing [18]. Mauri et al. [19] demonstrated that when flow rate was increased, there was no simultaneous increase in the ROX index, indicating a more critical condition in the patient. Therefore, in patients treated with HFNC, an increase in flow rate may serve as a reference strategy for the early identification of HFNC failure.In clinical practice, altered consciousness is one of the criteria for endotracheal intubation, and is considered a high-risk factor for early endotracheal intubation [20–22]. The GCS is the most commonly used index to quantify the degree of consciousness; the lower the GCS, the greater the degree of impaired consciousness. Patients with decreased levels of consciousness may lose the ability to protect their airway, thus increasing the risk of aspiration. Additionally, patients with a low level of consciousness are usually accompanied by decreased voluntary respiratory function and reduced ability to cooperate with noninvasive oxygen therapy support. Furthermore, BMI is a factor that influences the failure of HFNC [23, 24]. Obese patients typically exhibit low lung compliance and high airway resistance, which can complicate the ventilation process [25]. This Preliminary risk stratification model effectively combines the ROX index, GCS, flow rate, and BMI, offering greater discriminatory power and intuitive use compared to a single ROX index. However, it remains an exploratory tool. Although the internal validation results are promising, the Preliminary risk stratification model is currently only suitable for Preliminary risk stratification and should not be used directly for clinical decision-making until it has been validated in an external, independent cohort.

Of note, the ROX index used in this model refers to the initial value at HFNC initiation, with an optimal cutoff of 5.32. Although the ROX-12 h measurement demonstrated superior discriminative ability (AUC 0.721 vs. 0.613 for ROX-initial), the preliminary risk stratification model was intentionally developed using the initial ROX value for the following reasons. First, the primary clinical utility of a risk stratification tool lies in its ability to inform decision-making as early as possible. A model relying on 12-hour data would inevitably delay the decision window, potentially missing the opportunity for timely intervention. Second, the initial ROX value is available immediately upon HFNC initiation, making it more practical for bedside application in emergency and ICU settings. Third, while the 12-hour ROX provides better prognostic discrimination, it can be used as a dynamic reassessment tool after initial stratification—patients with low initial ROX can be monitored more closely, and their 12-hour ROX can further refine the risk estimate. Therefore, the model using ROX-initial serves as an entry-level screening tool, while the 12-hour ROX cutoff of 6.95 provides additional prognostic refinement for patients who remain on HFNC.

The use of HFNC has become increasingly prevalent in sepsis patients. A growing number of patients are being treated with HFNC; however, a significant portion will fail and subsequently require IMV [26]. Timely prediction of the likelihood of HFNC failure is crucial to mitigate adverse outcomes due to delayed intubation and IMV. Due to its effects on gas exchange and respiratory mechanics, a possible delay in intubation and IMV was observed because the deterioration in the clinic picture could be masked [9, 27]. Delayed intubation in patients with failed HFNC treatment may lead to increased mortality [28, 29]. These observations are consistent with our findings. Our findings show that among patients who failed HFNC, those delaying intubation for longer than 12 h had numerically higher in-hospital mortality compared to those intubated within 12 h (1.476-fold for 12–48 h and 1.566-fold for > 48 h); however, these differences did not reach statistical significance. Despite the lack of statistical significance, the numerical trend suggests a potential risk associated with delayed intubation. Given that early identification of HFNC failure remains clinically important regardless of the non-significant mortality difference, we recommend close monitoring and prompt preparation for intubation within the early phase (ideally within 12 h) after HFNC initiation. Future large-scale prospective studies are warranted to confirm the relationship between delayed intubation and mortality in this population.

This study utilized a large clinical database to validate the utility of the ROX index in patients with sepsis and established a clinically applicable Preliminary Risk Stratification Model. However, this study has certain limitations. First, as a single-center, retrospective analysis, this study is prone to methodological flaws and biases. Of particular note is clinical decision-making bias: the study defined HFNC failure as “the need for intubation and IMV,” which is essentially an operational endpoint based on clinicians’ comprehensive assessment rather than a fully objective physiological endpoint. Therefore, our definition of the outcome may, to some extent, reflect clinical decision-making patterns rather than purely physiological failure of HFNC. The results of this study should be interpreted as reflecting “the association between the ROX index and the risk of HFNC failure in a real-world clinical decision-making setting.” To assess and mitigate the impact of the aforementioned biases on the study’s conclusions, we conducted two sensitivity analyses: PSM to adjust for baseline confounders: To isolate the effect of “disease severity” on clinicians’ intubation decisions, we performed PSM to balance baseline severity characteristics (e.g., demographics, critical illness scoring systems) between the HFNC success and failure groups. In the PSM cohort, the AUC of the ROX-12 h for predicting HFNC failure was 0.707 (95% CI: 0.658–0.784), which was highly consistent with the main analysis (AUC = 0.721). Expanding the outcome definition reduces selection bias: Considering that some clinicians tend to attempt NIV as a “last conservative treatment” before intubation rather than proceeding directly to intubation, we redefined the outcome as “escalation of respiratory support (receiving NIV or IMV following HFNC failure)” and re-included 43 patients who received NIV (HFNC-NIV-IMV) for a sensitivity analysis. The results showed that the AUC for ROX-12 h was 0.710 (95% CI: 0.652–0.768), which was highly consistent with the primary analysis. Taken together, the two sensitivity analyses demonstrate that, although decision-making bias inherent in the retrospective design cannot be completely eliminated, the high stability of the results strongly supports the robust predictive value of the ROX index in the real-world management of sepsis patients receiving HFNC. Second, the findings of this study have not yet been externally validated and cannot be directly applied in clinical practice. The development of clinical scales requires prospective designs and independent validation cohorts. Therefore, this model serves solely as a framework for generating hypotheses for future prospective studies, rather than as a clinical tool at this stage; further validation through multicenter, prospective studies is still needed.

## Conclusion

In this retrospective cohort study of patients with sepsis, the ROX index demonstrated incremental predictive value for HFNC failure within the first 12 h of treatment. In particular, when evaluated in conjunction with a Preliminary risk stratification model incorporating the GCS, BMI, and initial flow rate, it improved predictive accuracy and could serve as a warning indicator of impending HFNC failure. Given the retrospective design of this study, these findings require prospective validation before clinical application.

## Supplementary Information

Below is the link to the electronic supplementary material.

Supplementary Material 1

Supplementary Material 2

Supplementary Material 3

Supplementary Material 4

Supplementary Material 5

Supplementary Material 6

Supplementary Material 7

Supplementary Material 8

Supplementary Material 9

Supplementary Material 10

Supplementary Material 11

Supplementary Material 12

## Data Availability

Data will be made available on request. For requesting data, please write to the corresponding author.

## Abbreviations

HFNC: High-Flow Nasal Cannula
ROX: Respiratory Rate-Oxygenation (Index)
MIMIC: Medical Information Mart for Intensive Care
BIDMC: Beth Israel Deaconess Medical Center
MIT: Massachusetts Institute of Technology
SOFA: Sequential Organ Failure Assessment
NIV: Non-Invasive Ventilation
IMV: Invasive Mechanical Ventilation
SpO₂: Pulse Oxygen Saturation
FiO₂: Fraction of Inspired Oxygen
RR: Respiratory Rate
BMI: Body Mass Index
SBP: Systolic Blood Pressure
DBP: Diastolic Blood Pressure
MAP: Mean Arterial Pressure
HR: Heart Rate
CCI: Charlson Comorbidity Index
GCS: Glasgow Coma Scale
SAPS II: Simplified Acute Physiology Score II
MELD: Model for End-Stage Liver Disease
CRRT: Continuous Renal Replacement Therapy
ROC: Receiver Operating Characteristic
AUC: Area Under the Curve
OR: Odds Ratio
CI: Confidence Interval
KM: Kaplan-Meier
ICU: Intensive Care Unit
POX-HR: Pulse Oxygenation-Heart Rate (Index)
MI: Multiple Imputation
PSM: Propensity Score Matching
