# Supplementary material for: Prediction of high-flow nasal cannula failure in patients with sepsis by a preliminary risk stratification model based on the ROX index
Source: BMC Infect Dis. 2026 Aug 3;26:1693. doi: 10.1186/s12879-026-14135-3 (PMC13595640; doi:10.1186/s12879-026-14135-3)
Supplement: Supplementary file 1 [file 12879_2026_14135_MOESM10_ESM.docx]

Table S5 The process of assigning scores to each risk factor

| Factor | β | Categories | REFERENCE VALUE  (W_ij_) | β*（W_ij-_W_iREF_） | SCORE  β（W_ij-_W_iREF_）/B |
| --- | --- | --- | --- | --- | --- |
| GCS | -0.253 | 13-15 | W_iREF_ =14 | 0 | 0 |
|  |  | 9-12 | 10.5 | 0.8855 | 2 |
|  |  | 6-8 | 7 | 1.771 | 4 |
|  |  | 3-5 | 4 | 2.53 | 6 |
| BMI | 0.065 | <18.5 | 16 | -0.3705 | -1 |
|  |  | 18.5-24.9 | W_iREF_ =21.7 | 0 | 0 |
|  |  | 25-30 | 27.5 | 0.377 | 1 |
|  |  | >30 | 38.75 | 1.1083 | 3 |
| Flow rate | 0.029 | 1-25 | W_iREF_ =13 | 0 | 0 |
|  |  | 26-50 | 38 | 0.725 | 2 |
|  |  | 51-75 | 63 | 1.45 | 3 |
|  |  | 76-100 | 88 | 2.175 | 5 |
| ROX-initial | -0.140 | ≥5.32 | W_iREF_ =9.8029 | 0 | 0 |
|  |  | <5.32 | 3.650 | 0.8614 | 2 |
