# Supplementary material for: Prediction of high-flow nasal cannula failure in patients with sepsis by a preliminary risk stratification model based on the ROX index
Source: BMC Infect Dis. 2026 Aug 3;26:1693. doi: 10.1186/s12879-026-14135-3 (PMC13595640; doi:10.1186/s12879-026-14135-3)
Supplement: Supplementary file 2 [file 12879_2026_14135_MOESM11_ESM.docx]

Table S6 Risks Associated with Score Totals

| Score Totals | Estimate of Risk | Score Totals | Estimate of Risk |
| --- | --- | --- | --- |
| -1 | 0.7% | 8 | 27.1% |
| 0 | 1.1% | 9 | 36.6% |
| 1 | 1.6% | 10 | 47.3% |
| 2 | 2.5% | 11 | 58.3% |
| 3 | 3.9% | 12 | 68.6% |
| 4 | 5.9% | 13 | 77.3% |
| 5 | 8.9% | 14 | 84.1% |
| 6 | 13.3% | 15 | 89.2% |
| 7 | 19.2% | 16 | 92.8% |
