# Supplementary material for: Prediction of high-flow nasal cannula failure in patients with sepsis by a preliminary risk stratification model based on the ROX index
Source: BMC Infect Dis. 2026 Aug 3;26:1693. doi: 10.1186/s12879-026-14135-3 (PMC13595640; doi:10.1186/s12879-026-14135-3)
Supplement: Supplementary file 3 [file 12879_2026_14135_MOESM1_ESM.docx]

Patients with sepsis documented in the MIMIC-IV using HFNC N=1679

Excluded the following patients(N=1363) multiple hospitalizations(N=612) hospital stay less than 48h(N=27) conditions with high risk of hypercapnia such as COPD(N=378) tracheostomy performed before admission(N=9) HFNC not used before intubation, but used after intubation(N=275) records of non-invasive ventilation use before intubation(N=43) HFNC for less than 2h(N=9) lack of ROX-related data(N=10)

Patients for the final analysis N=316

Patients with HFNC success N=200

Patients with HFNC failure N=116

Figure S1 Flow chart of patient selection from the MIMIC-IV database. After applying the inclusion and exclusion criteria, 316 patients were finally included in the analysis, of whom 200 were classified into the HFNC success group and 116 into the HFNC failure group.
