# Supplementary material for: Prediction of high-flow nasal cannula failure in patients with sepsis by a preliminary risk stratification model based on the ROX index
Source: BMC Infect Dis. 2026 Aug 3;26:1693. doi: 10.1186/s12879-026-14135-3 (PMC13595640; doi:10.1186/s12879-026-14135-3)
Supplement: Supplementary file 4 [file 12879_2026_14135_MOESM2_ESM.docx]

Figure S2 Kaplan-Meier survival curve for HFNC success in sepsis patients stratified by ROX-12h index (Group 1<6.95; Group 2≥6.95). Log-rank P < 0.001.
