# Supplementary material for: Prediction of high-flow nasal cannula failure in patients with sepsis by a preliminary risk stratification model based on the ROX index
Source: BMC Infect Dis. 2026 Aug 3;26:1693. doi: 10.1186/s12879-026-14135-3 (PMC13595640; doi:10.1186/s12879-026-14135-3)
Supplement: Supplementary file 8 [file 12879_2026_14135_MOESM6_ESM.docx]

Table S1 Multivariable logistic regression analysis of risk factors for HFNC failure

|  | β | S.E | Wald | P | OR | 95%CI |
| --- | --- | --- | --- | --- | --- | --- |
| GCS | -0.253 | 0.040 | 40.968 | 0.000 | 0.776 | 0.718-0.839 |
| BMI | 0.065 | 0.024 | 7.244 | 0.007 | 1.067 | 1.018-1.119 |
| ROX-initial | -0.140 | 0.054 | 6.605 | 0.010 | 0.869 | 0.781-0.967 |
| Flow rate | 0.029 | 0.010 | 9.084 | 0.003 | 1.029 | 1.010 -1.049 |
| Intercept | -1.407 |  |  |  |  |  |
