# Supplementary material for: Prediction of high-flow nasal cannula failure in patients with sepsis by a preliminary risk stratification model based on the ROX index
Source: BMC Infect Dis. 2026 Aug 3;26:1693. doi: 10.1186/s12879-026-14135-3 (PMC13595640; doi:10.1186/s12879-026-14135-3)
Supplement: Supplementary file 9 [file 12879_2026_14135_MOESM7_ESM.docx]

Table S2 The discriminatory value of ROX and Preliminary Risk Stratification Model to assess HFNC failure

|  | Cut-off | accuracy (%) | Se (%) | Sp (%) | PPV (%) | NPV (%) |
| --- | --- | --- | --- | --- | --- | --- |
| ROX-initial | 5.32 | 61.4 | 59.5 | 62.5 | 47.9 | 72.7 |
| ROX-2h | 5.25 | 62.7 | 51.7 | 69.0 | 49.2 | 71.1 |
| ROX-6h | 5.69 | 68.4 | 62.2 | 71.4 | 51.7 | 79.3 |
| ROX-12h | 6.95 | 67.9 | 72.5 | 66.0 | 46.4 | 85.5 |
|  | 5.22 | 69.7 | 42.5 | 80.7 | 47.2 | 77.6 |
|  | 8.05 | 60.6 | 80.0 | 52.8 | 40.8 | 86.7 |
| Preliminary Risk Stratification Model | 5.5 | 75.6 | 77.5 | 72.4 | 82.9 | 65.1 |
|  | 6 | 72.5 | 80.0 | 59.5 | 77.3 | 63.3 |
|  | 5 | 69.6 | 63.5 | 80.2 | 84.7 | 56.0 |

Definition of abbreviations: NPV = negative predictive value; PPV = positive predictive value; Se = sensitivity; Sp = specificity
