# Supplementary material for: Prediction of high-flow nasal cannula failure in patients with sepsis by a preliminary risk stratification model based on the ROX index
Source: BMC Infect Dis. 2026 Aug 3;26:1693. doi: 10.1186/s12879-026-14135-3 (PMC13595640; doi:10.1186/s12879-026-14135-3)
Supplement: Supplementary file 10 [file 12879_2026_14135_MOESM8_ESM.docx]

Table S3 Logistic regression model analysis of the relationship between ROX and HFNC Failure.

| Model 1 | *OR* | 95%*CI* | *P* | Model 2 | *OR* | 95%*CI* | *P* |
| --- | --- | --- | --- | --- | --- | --- | --- |
| ROX≥6.95 | REF. |  |  | ROX≥6.95 | REF. |  |  |
| ROX<6.95 | 5.115 | 2.886~9.068 | <0.05 | ROX<6.95 | 4.968 | 2.780~8.876 | <0.05 |
| Model 3 | *OR* | 95%*CI* | *P* | Model 4 | *OR* | 95%*CI* | *P* |
| ROX≥6.95 | REF. |  |  | ROX≥6.95 | REF. |  |  |
| ROX<6.95 | 5.729 | 3.079~10.661 | <0.05 | ROX<6.95 | 5.780 | 2.839~11.771 | <0.05 |

Note: Model I: Unadjusted for confounding factors. Model II: Adjusted for confounding factors—demographic data such as age and gender. Model III: Adjusted for confounding factors—demographic data such as age and gender and vital signs. Model IV: Adjusted for confounding factors—demographic data such as age and gender, vital signs, Charlson Comorbidity Index and Critical illness scoring systems. REF: Reference group.
