# Supplementary material for: Prediction of high-flow nasal cannula failure in patients with sepsis by a preliminary risk stratification model based on the ROX index
Source: BMC Infect Dis. 2026 Aug 3;26:1693. doi: 10.1186/s12879-026-14135-3 (PMC13595640; doi:10.1186/s12879-026-14135-3)
Supplement: Supplementary file 11 [file 12879_2026_14135_MOESM9_ESM.docx]

Table S4 Subgroup analysis of the relationship between ROX and HFNC failure

| Characteristics | N | ROX≥6.95 | ROX<6.95 |  |
| --- | --- | --- | --- | --- |
|  |  |  | OR (95% CI) | P |
| Age |  |  |  |  |
| <65 | 123 | Ref | 7.39(3.20-17.05) | 0.000 |
| ≥65 | 154 | Ref | 3.72 (1.66 ~ 8.32) | 0.001 |
| Gender |  |  |  |  |
| Male | 161 | Ref | 7.71 (3.55 ~ 16.72) | 0.000 |
| Female | 116 | Ref | 2.60 (1.06 ~ 6.33) | 0.036 |
| BMI |  |  |  |  |
| <26 | 135 | Ref | 5.87 (2.41 ~ 14.30) | 0.000 |
| ≥26 | 142 | Ref | 4.79 (2.24 ~ 10.25) | 0.000 |
| Ethnicity |  |  |  |  |
| White | 207 | Ref | 5.38 (2.78 ~ 10.41) | 0.000 |
| Other | 70 | Ref | 4.50 (1.41 ~ 14.35) | 0.011 |
| HR |  |  |  |  |
| <95 | 135 | Ref | 6.66 (2.70 ~ 16.41) | 0.000 |
| ≥95 | 142 | Ref | 4.09 (1.93 ~ 8.67) | 0.000 |
| SBP |  |  |  |  |
| <120 | 146 | Ref | 5.58 (2.39 ~ 13.03) | 0.000 |
| ≥120 | 131 | Ref | 4.95 (2.24 ~ 10.90) | 0.000 |
| DBP |  |  |  |  |
| <65 | 127 | Ref | 4.80 (2.04 ~ 11.28) | 0.000 |
| ≥65 | 150 | Ref | 5.35 (2.47 ~ 11.58) | 0.000 |
| MBP |  |  |  |  |
| <80 | 134 | Ref | 11.29 (3.99 ~ 31.96) | 0.000 |
| ≥80 | 143 | Ref | 3.27 (1.58 ~ 6.76) | 0.001 |
| RR |  |  |  |  |
| <24 | 136 | Ref | 6.58 (2.80 ~ 15.49) | 0.000 |
| ≥24 | 141 | Ref | 3.88 (1.77 ~ 8.53) | 0.000 |
| Spo2 |  |  |  |  |
| <95 | 137 | Ref | 6.30 (2.63 ~ 15.09) | 0.000 |
| ≥95 | 140 | Ref | 4.43 (2.03 ~ 9.71) | 0.000 |
| Flow rate-initial |  |  |  |  |
| <30 | 106 | Ref | 5.48 (1.96 ~ 15.34) | 0.001 |
| ≥30 | 171 | Ref | 5.07 (2.52 ~ 10.18) | 0.000 |
| ROX-initial |  |  |  |  |
| <5.70 | 134 | Ref | 5.34 (2.16 ~ 13.21) | 0.000 |
| ≥5.70 | 143 | Ref | 4.58 (1.99 ~ 10.58) | 0.000 |
| SOFA |  |  |  |  |
| <6 | 136 | Ref | 7.40 (2.74 ~ 19.96) | 0.000 |
| ≥6 | 141 | Ref | 4.05 (1.95 ~ 8.40) | 0.000 |
| SAPS-II |  |  |  |  |
| <36 | 133 | Ref | 7.25 (2.98 ~ 17.66) | 0.000 |
| ≥36 | 144 | Ref | 3.91 (1.83 ~ 8.34) | 0.000 |
| Charlson Comorbidity Index |  |  |  |  |
| <6 | 122 | Ref | 7.00 (2.95 ~ 16.63) | 0.000 |
| ≥6 | 155 | Ref | 3.51 (1.60 ~ 7.70) | 0.002 |
| GCS |  |  |  |  |
| <13 | 106 | Ref | 3.54 (1.59 ~ 7.91) | 0.002 |
| ≥13 | 171 | Ref | 8.00 (3.05 ~ 20.99) | 0.000 |
| Meld |  |  |  |  |
| <13 | 126 | Ref | 4.31 (1.72 ~ 10.77) | 0.002 |
| ≥13 | 151 | Ref | 5.95 (2.82 ~ 12.55) | 0.000 |
| CRRT |  |  |  |  |
| NO | 248 | Ref | 6.38 (3.27 ~ 12.44) | 0.000 |
| YES | 29 | Ref | 5.62 (0.94 ~ 33.76) | 0.059 |
| Vasopressor use |  |  |  |  |
| NO | 173 | Ref | 3.13 (1.18 ~ 8.30) | 0.022 |
| YES | 104 | Ref | 10.20 (4.08 ~ 25.49) | 0.000 |
| Length of hospital stay |  |  |  |  |
| <14 | 147 | Ref | 14.43 (3.18 ~ 65.57) | 0.000 |
| ≥14 | 130 | Ref | 4.70 (2.24 ~ 9.85) | 0.000 |
| Length of ICU stay |  |  |  |  |
| <7 | 163 | Ref | 15.25 (1.86 ~ 125.15) | 0.011 |
| ≥7 | 114 | Ref | 4.93 (2.18 ~ 11.16) | 0.000 |
| Duration of HFNC |  |  |  |  |
| <24 | 112 | Ref | 10.30 (4.13 ~ 25.67) | 0.000 |
| ≥24 | 165 | Ref | 3.79 (1.75 ~ 8.20) | 0.000 |
