# Supplementary material for: Prediction of high-flow nasal cannula failure in patients with sepsis by a preliminary risk stratification model based on the ROX index
Source: BMC Infect Dis. 2026 Aug 3;26:1693. doi: 10.1186/s12879-026-14135-3 (PMC13595640; doi:10.1186/s12879-026-14135-3)
Supplement: Supplementary file 12 [file 12879_2026_14135_MOESM4_ESM.docx]

Figure S4 ROC curve of ROX (expanded cohort, n=359)
