# Supplementary material for: Prediction of high-flow nasal cannula failure in patients with sepsis by a preliminary risk stratification model based on the ROX index
Source: BMC Infect Dis. 2026 Aug 3;26:1693. doi: 10.1186/s12879-026-14135-3 (PMC13595640; doi:10.1186/s12879-026-14135-3)
Supplement: Supplementary file 14 [file 12879_2026_14135_MOESM12_ESM.doc]

Table S7 Missing number (%) for characteristics

| **Characteristics** | **Missing number (%)** |
| --- | --- |
| **Demographic** |  |
| Age | 0(0) |
| Male, gender | 0(0) |
| Ethnicity, White | 0(0) |
| Height | 76(24.05%) |
| Weight | 15(4.75%) |
| **Vital sign** |  |
| HR | 0(0) |
| SBP | 0(0) |
| DBP | 0(0) |
| MBP | 0(0) |
| RR | 0(0) |
| SpO2 | 0(0) |
| Flow rate-initial | 0(0) |
| **Critical illness scoring systems** |  |
| SOFA-first-day | 0(0) |
| GCS-first-day | 0(0) |
| SAPS-II-first-day | 15(4.75%) |
| Meld-first-day | 24(7.59%) |
| Charlson Comorbidity Index | 0(0) |
| **ROX** |  |
| ROX-initial | 0(0) |
| ROX-2h | 0(0) |
| ROX-6h | 19(6.01%) |
| ROX-12h | 39(12.34%) |
| **Clinical treatment information** |  |
| CRRT | 0(0) |
| Vasopressor use | 0(0) |
| Length of hospital stay | 0(0) |
| Length of ICU stay | 0(0) |
| Duration of HFNC | 0(0) |
